# Decreasing the proportion of conflict does not help to exploit congruency cues in a Stroop task

**DOI:** 10.3389/fcogn.2024.1452711

**Published:** 2024-12-04

**Authors:** Luis Jiménez, David Gallego, María José Lorda, Cástor Méndez

**Affiliations:** ^1^IPsiUS and Facultad de Psicología, Universidad de Santiago de Compostela, Santiago de Compostela, Spain; ^2^Universidad de La Rioja (UNIR), Logroño, Spain

**Keywords:** Stroop, cognitive control, learning, congruency cueing, interference tasks

## Abstract

**Introduction:**

Humans are able to regulate the intensity with which they exert cognitive control in interference tasks in terms of factors such as the control level required on the previous trial, and the overall frequency of conflict. However, recent research has shown that the ability to follow explicit cues predicting the required level of control is more limited than previously assumed. Specifically, participants in color Stroop tasks did only take advantage of pre-cues informing them about the congruency of the following trial when the cue was presented in the interval between successive trials, but not when the information was conveyed by the preceding trial.

**Method:**

Here we explore the boundary conditions of these sequential cueing effects by using a Stroop task in which the proportion of high-conflict trials was increased, to improve practice with the rules, or decreased, to make the task less demanding.

**Results:**

The results showed no effect of trial-by-trial cueing, neither increasing nor decreasing the proportion of high-conflict trials. Furthermore, the cueing effect was not observed either when the cue was conveyed by neutral trials, thus reducing the conflation between the conflict present on a trial and the conflict that this trial predicts.

**Discussion:**

As a whole, the results illustrate how difficult it is to adjust control parameters on the fly on the basis of sequential cues, even if they are explicit.

## Cognitive control

Cognitive control refers to a set of mechanisms by which humans focus their processing on what is relevant to their current goals, while resisting the interference coming from more habitual and automatic tendencies (Cohen, 2017). The functions of cognitive control are generally adaptive, in that they allow people, for instance, to drive home without getting distracted by stimuli that call their attention off the road. However, these mechanisms must be flexibly guided by their previous experience, as conditions vary widely in terms of the potential usefulness of the information provided by the context to their goals. In the driving case, for instance, the amount of information available on the sides of the road depends on whether the driver is steering through a crowdy street or on a desert freeway. Fortunately, under certain conditions the right amount of control can be explicitly conveyed by signs designed to inform people about the need for control, such as flickering lights, signs of school zone, or other warning signs.

In the lab, the modulation of cognitive control has been studied by using simplified interference tasks in which participants respond to stimuli composed of two types of features: target features containing the relevant information for responding, and distractor features which are nominally irrelevant, but contain information associated with the relevant targets. In the case of the Stroop task (Stroop, 1935) participants respond to the color in which a word is printed while ignoring its meaning, that may refer to the same or to a different color. The results of this paradigm have provided researchers with mounting evidence on how hard it is to ignore the information provided by such distractor features, as participants are slower and less accurate in responding to trials in which the color of the word is not congruent with its meaning (MacLeod, 1992). Variations of this task have been used to investigate how the previous experience modulates this congruency effect, depending on global statistics such as the proportion of incongruent trials presented over a given context (Crump et al., 2006; Logan and Zbrodoff, 1979), and on more specific factors such as whether the previous trial was incongruent (Kerns et al., 2004), or whether the congruency of each trial was signaled by a predictive cue (Bugg and Smallwood, 2016; Jiménez et al., 2020, 2021; Jiménez and Méndez, 2013, 2014).

To account for these dynamics of cognitive control, Braver (2012) proposed a dual model which assumed that control functions are regulated in two operating modes: proactively, in a strategic and sustained manner that depends on global features such as the overall proportion of conflict trials, and reactively, in a just-in-time manner that responds to specific conditions observed within a single trial. In contrast, the conflict monitoring hypothesis (Botvinick et al., 2001) provided a unitary account to both phenomena, assuming that conflict monitoring and the subsequent adaptation to conflict could be produced automatically on each trial, resulting both in the specific adjustments produced after each trial, and in the gradual trend that gets accumulated with practice across trials. More recently, however, these authors reassessed their assumption that the adaptation process could be exerted automatically, and argued in favor of the inherent costs of cognitive control (Shenhav et al., 2013), assuming that control regulation will rely on a strategic decision that depends on its expected value. Finally, another group of theories attributed these dynamics to the general effects of associative learning on performance, proposing that the repeated presentation of the same control demands under a given context would lead to an association between that context and the control response, hence releasing the same control response whenever this context reappears (Abrahamse et al., 2016; Chiu and Egner, 2019; Egner, 2014).

## Cueing control

If a control response can become associated to a context, then the question arises as to whether this associative process could also work in a predictive way, allowing agents to use predictive cues to help them to prepare for an upcoming conflict. This question has been less widely explored, even though it has important bearings on the characterization of these control dynamics and on any applied technology aimed to optimize performance. For instance, if control regulation depends on a strategic decision that requires an explicit preparation, then it should be promoted by allowing both temporal and conceptual separation between cues and targets. In contrast, if control regulation arises as the outcome of automatic and associative learning processes, then the vicinity between cues and targets might benefit performance, as contiguity is commonly conceived as the main precondition of associative learning (Boakes and Costa, 2014). Moreover, this cueing role could be played by an external signal, or by the features of the preceding trial, as it is often observed in the context of sequence learning paradigms (e.g., D'Angelo et al., 2013; Jiménez et al., 2020; Jiménez and Vázquez, 2008).

Earlier studies that addressed the question of whether participants can learn to use cues to prepare for the amount of control needed to respond to the forthcoming trial provided some positive results (Ghinescu et al., 2010; Gratton et al., 1992; Logan and Zbrodoff, 1982), but they were also inconsistent in terms of whether the cues should be 100% valid or may contain just probabilistic information, and on whether the effects could be observed for both congruent and incongruent trials, or could be only helpful to respond to cued congruent trials (Aarts et al., 2008; Correa et al., 2009). Moreover, some studies just failed to obtain cueing benefits at all (Luks et al., 2007; van Driel et al., 2015), and some other questioned the interpretation of positive effects, which were mostly obtained in conditions involving two-choice trials. Wühr and Kunde (2008) suggested that these two-choice paradigms allowed participants to adopt a strategy opposite to that of controlling the processing of the distractor, involving either responding in terms of the distractor when the cue indicated a congruent successor, or against its identity when the cue warned about the presentation of an incongruent trial. Given that the distractor will always benefit responding to congruent trials, and that it can be useful to respond to incongruent trials in two-choice tasks, a convincing demonstration that congruency cues can be effectively used to regulate control would require demonstrating an advantage in responding to cued incongruent trials in conditions involving three or more choices.

One of the few studies satisfying these constraints was reported by Bugg and Smallwood (2016), using a four-choice Stroop task, where they reported that such cueing effects could be obtained for incongruent trials only under conditions involving long cue-to-stimulus intervals, and a long interval between successive trials. Moreover, a systematic exploration of the boundary conditions of such cueing effects by Jiménez et al. (2020, 2021) confirmed that these benefits were difficult to obtain, and that they were only observed on incongruent trials when the cues were presented in the interval between successive trials, but not when the information was conveyed by the preceding trial. They also showed that those benefits were obtained exclusively when (1) the cues were 100% reliable, (2) the effects were compared between blocks rather than intermixing cued and non-cued trials, and (3) the task required naming the target colors, rather than responding manually on arbitrary keys, arguably indicating that the cueing effect was easier to observe when participants' responses relied on overlearned associations rather than on arbitrary stimulus-response mapping.

This pattern of results seems more consistent with a characterization of the cueing effect as the product of a strategic decision requiring a sustained effort, rather than as the output of an automatic and associative learning process. For instance, the difference between verbal and manual tasks suggests that retrieving information about the arbitrary mapping between colors and responses may detract some cognitive resources needed to exploit the predictive contingencies. Moreover, the fact that these cueing effects are improved when the intervals between trials and between cues and targets increase also suggests that participants need time to act upon the cues, and that there is competition for resources between responding to the imperative task and exploiting the cueing information. Finally, even though the advantage of presenting the cues in the interval between trials might be taken as evidence in favor of the associative view if it suggests that closer cues are more effective, this is not consistent with the previous results showing that longer intervals between cues and targets results in stronger effects (Bugg and Smallwood, 2016). Rather, the difficulty of using the previous trial as a cue may point to the conflation between two simultaneous requirements of cognitive control that converge in these conditions, as participants would need to act upon the control demands imposed by a trial while they are also trying to extract the cueing information conveyed by that trial with respect to the conflict expected on the following one.

In this context, the goal of the present research is to assess whether it is possible to find control cueing effects in conditions in which the cueing information is provided by the preceding trial, manipulating the global amount of conflict in the task, and whether or not the cueing trials themselves are free of conflict. From a strategic view of this regulatory process, we reasoned that, if preparing for a high-conflict trial requires more cognitive resources than preparing for a low-conflict trial, and if these costs tend to accumulate over a block of practice, then reducing the proportion of incongruent trials could be a valid way to reduce the burden of following these cues. Correspondingly, Experiment 1 was designed as a replication of Experiment 8 from Jiménez et al. (2021), but we reduced the proportion of incongruent trials from 0.50 to 0.20. In contrast, from an associative view, if people need practice and repeated experience with the association between cues and outcomes to get used to exploit these rules (e.g., Braem et al., 2024), then increasing the occasions of practicing these rules could be a better way to improve their effects. To distinguish between these two opposite views, we compared two versions of the task in Experiment 2, one replicating the conditions arranged in Experiment 1, and the other inverting these conditions to increase the proportion of incongruent trials, thus increasing the practice with incongruency cues.

One consequence of the proposed designs is that using the previous color as the cue while simultaneously manipulating the proportion of congruency will affect the frequency of appearance of each color. For instance, in the condition of high proportion of congruency, the colors that predict a congruent successor will arise more frequently than those predicting an incongruent successor. Because we found some evidence suggesting that responding could become slower for those trials presented after infrequent trials, in an effect resembling the oddball effect (Barcelo et al., 2006; see also Notebaert et al., 2009), and because this effect could be confounded with the cueing effect, we considered the need to distinguish those effects by designing two additional experiments. In Experiments 3 and 4, we tested our initial hypothesis that reducing the frequency of high demanding trials could improve the efficiency of incongruency cues in conditions in which all colors were presented with the same likelihood, but in which only one color was predictive of high-conflict successors. In Experiment 4, the design also included a 50% of neutral trials as a way to assess whether the cueing effect could be more easily observed when the cueing information was conveyed by a non-conflictive trial, thus reducing the cognitive demands imposed by the informative trials and separating cueing from cued trials.

## Experiment 1

Experiment 1 was designed as a conceptual replication of Experiment 8 from Jiménez et al. (2021), using a four-choice vocal Stroop task, and comparing participants' performance on a cued phase composed of three consecutive blocks with that observed on a control phase composed of another three blocks of non-cued trials. In the original experiment, participants were informed that the color of each trial conveyed information about whether the next trial would be easy or difficult (i.e., congruent, or incongruent), but they showed no benefit from the congruency cues. Indeed, they responded slower to incongruent trials in cued blocks as compared to control blocks. We reasoned that, if following the rules was perceived as effortful, especially when preparing for a high-conflict trial, then reducing the amount of such trials could reduce the accumulated cost associated to acting upon the rules, as well as make these incongruency cues a bit more salient. Thus, we altered the balance between congruent and incongruent trials, arranging a condition of high proportion of congruency as a testbed for the utility of congruency cues.

### Method

#### Participants

This and the following experiments in the series were conducted in accordance with Spanish regulations, complying with the ethical standards of the 1964 Declaration of Helsinki. They were part of a research project approved by the local Ethics Committee of the University of Santiago de Compostela. Because the experiment followed Jiménez et al.'s (2021) Experiment 8, and was meant to be comparable with it, we relied on the same sample size. Thus, we tested 24 volunteers (20 female, M_age_ = 20, Range = 19–25) recruited from the University of Santiago de Compostela. Participants in this and the following experiments signed informed consents before participating. They were native speakers of Spanish, had normal or corrected-to-normal vision acuity, and normal color vision.

#### Apparatus and stimuli

The experiment was designed and controlled using INQUISIT 4 (Millisecond_Software, 2015) software, running on personal computers connected to 22-inch monitors with a resolution of 1,920 × 1,080 pixels. Participants viewed the monitors from an unrestricted distance of ~60 cm. On each trial, they were presented with a Spanish word referring to one of the following colors: red (“rojo”), blue (“azul”), green (“verde”), and yellow (“amarillo”). All the words were printed in Arial, lower case, 32-point font, against a gray background, and could be printed in one of the four different colors (red, green, blue, or yellow). Participants responded vocally, naming the color in which the words were printed, and responses were detected and recognized by the system, using the voice key and the speech recognition modules built in INQUISIT 4 software.

#### Procedure

After initial instructions, participants were informed that they would see words printed either in red, blue, green, or yellow, and that their task was to name the color in which the words were printed, responding as fast and accurately as possible. To get used to the task, and to train the system to recognize participants' voices, the procedure started with a practice block in which participants responded to 50 trials using four neutral words, house (“casa”), car (“coche”), plant (“planta”) and zone (“zona”) printed randomly in any of these four colors.

After the practice block, participants completed six experimental blocks, each composed of 96 trials. These blocks were divided in two phases, corresponding to cued and control phases. Even-numbered participants were first presented with the control phase, followed by the cued phase, and odd-numbered participants were trained in the opposite order. The predictive instructions were presented before the start of the cueing phase, and the information about the specific contingencies was repeated in the intervals between successive blocks. Participants were also informed about the switch between phases. Therefore, in advance to Block 4, participants who had been trained with the cued blocks were informed that the cues would no longer be informative, whereas those who were first presented with the control phase were informed at this point about which colors predicted “easy” (i.e., congruent) or “difficult” (i.e., incongruent) successors.

During the cued phase, the congruency of every trial was reliably predicted by the preceding target color. Two colors were assigned for each participant as predictors of a congruent successor, and the other two were selected as predictors of an incongruent successor. As 80% of the trials were congruent, the two colors predicting a congruent successor had a chance of .80 of being selected on each trial, while the two colors assigned as predictors of incongruent successors were selected with a probability of .20. During the cued blocks, each color was deterministically followed by the corresponding type of trial (i.e., congruent or incongruent), whereas during the control blocks these predictive contingencies were removed, but the same biased frequencies were maintained. Thus, congruent trials still appeared on 80% of the trials over the control blocks, and those colors assigned as predictors of such congruent trials were also chosen more frequently, even though they were no longer associated to the congruency of the successor. For each participant, two different colors acted as cues for congruent successors, and the remaining two predicted incongruent successors. The assignment of colors to cue values was counterbalanced between participants to make sure that all possible pairs acted equally often as cues for congruent and incongruent successors. This resulted in six different arrangements, each presented to four participants (see Table 1).

**Table 1:** Assignment of colors to predictive values for each counterbalance group for Experiments 1 and 2.

	**Predict congruent**		**Predict incongruent**	
Group 1	Red	Green	Blue	Yellow
Group 2	Blue	Yellow	Red	Green
Group 3	Red	Yellow	Green	Blue
Group 4	Green	Blue	Red	Yellow
Group 5	Red	Blue	Green	Yellow
Group 6	Green	Yellow	Red	Blue

Each trial started with a fixation cross presented at the center of the screen, which was replaced after 750 ms by the distractor word presented in the target color, which remained on screen until response. When an error was committed, this was marked by the word “error” written in black over a white screen during 1,000 ms. After a correct response, the next trial followed immediately. After each block, participants were informed about their average reaction time and percentage of correct responses, and they were asked to keep responding as fast as possible while maintaining a hit rate higher than .90.

#### Design

The design includes two main within-participants factors, *cueing* (cued vs. control phases) and *congruency* (congruent vs. incongruent trials). In addition, because the effect of cueing could be expected to differ depending on whether participants were first exposed to the cueing or to the control phase, we added *order* (cueing phase first vs. control phase first) as an additional between-participants variable in the main design. Previous experiments by Jiménez et al. (2021) also included *practice* (i.e., block of trials) as a third within-participants factor, assuming that it may take some time for participants to learn to exploit the cues. However, because the number of cued incongruent trials was reduced in this paradigm due to the unbalanced proportion of congruency, we removed this factor and assessed the cueing effect by comparing the whole sample of cued vs. control trials. Moreover, because presenting the same proportion of congruency and the same color frequencies on cued and control blocks would result in a large number of trials conforming to the rules, even during the control blocks, we distinguished within the control blocks between trials that complied with the rules, and those that contravened the rules. Specifically, if congruent trials were selected on 80% of the trials, and if the colors that acted as a cue for congruent successors also appeared on 80% of the trials, one might expect that congruent successors conforming to the rules would arise by chance in 64% (.80 × .80) of the control trials, or about 182 trials out of the 285 that composed the relevant trials within the control blocks. Moreover, an additional 4%, or about 11 trials, would correspond to incongruent trials generated according to the rules (.20 × .20). This would leave us with about 92 trials (74 congruent and 18 incongruent) that could be considered as control trials contravening the rules. In these conditions, comparing the overall responses to control and cueing trials as a whole could not be a good strategy to assess the cueing effects, especially if participants keep acting on these cues even after being told that the rules were no longer applicable (see Abrahamse et al., 2022). Instead, the evidence of cueing was inferred by comparing responding to cueing blocks and responding selectively to those control trials which did not conform to the cueing rules. In sum, the main cueing effects would be inferred by the results of two mixed-factors ANOVAs, taking reaction time (RTs) and percentage of correct responses as dependent variables, including *order* as a between-participants factor, and using *cueing* and *congruency* as within-participants variables. Even though our focus was mainly on the measures of latency, percentages of correct responses were also analyzed to confirm that any effect of cueing on RTs was not due to a tradeoff between speed and accuracy.

Finally, to assess whether performance over the control phase may reflect any impact from the cueing arrangements, either due to the earlier presentation of the cueing phase in half of the participants, or to any other effect dependent on the frequency structure of trials, two additional ANOVAs were conducted to compare RTs and percentage of hits over the control trials, using *order* as a between-participants factor, and *congruency* and *compliance with the training rules* (conforming vs. contravening) as within-participants variables.

#### Transparency and openness

All data from this and the remaining experiments are publicly available at the *Open Science Foundation*, together with the Inquisit code wrote to control these experiments, and the analyses conducted on JASP. Interested readers can retrieve this material at https://osf.io/xgkjr/.

### Results

**Figure 1:**
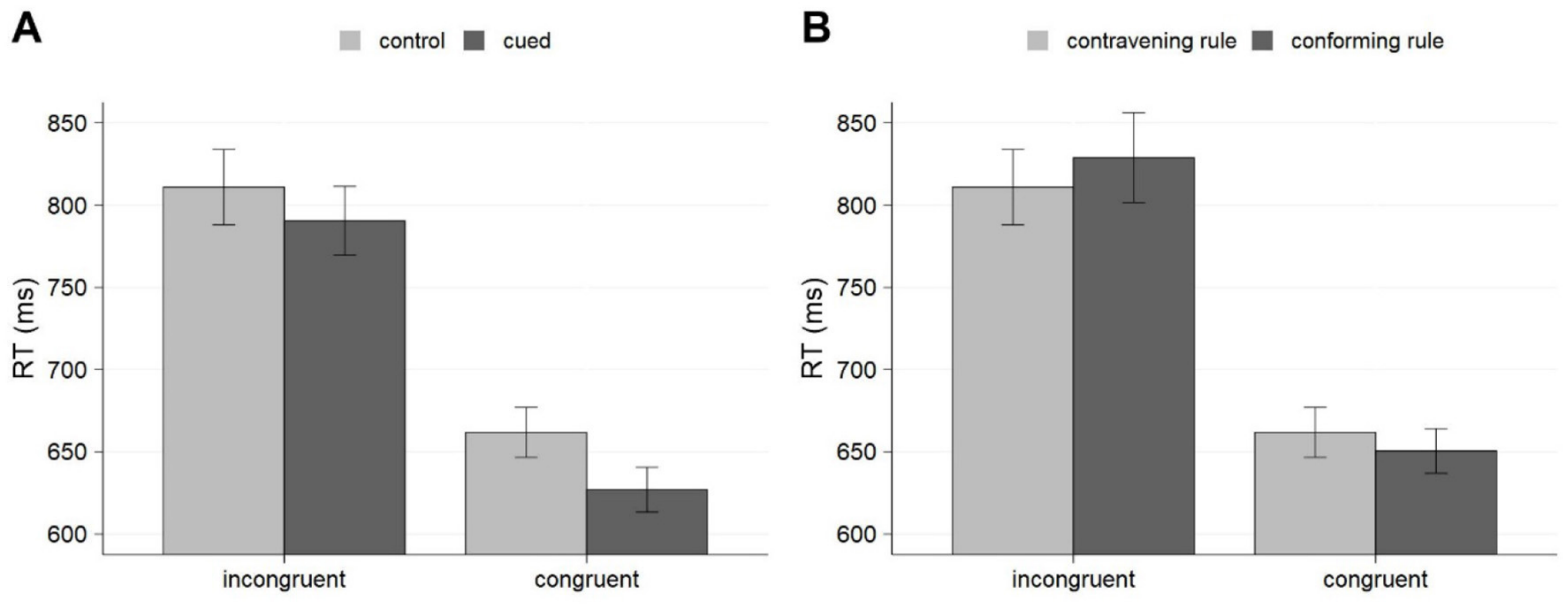
Mean RTs for cueing effects **(A)** and the presence vs. absence of the predictive rule on control blocks **(B)** for Experiment 1. Error bars represent the standard error of the mean.

To analyze RTs, we excluded the first trial from each block (1.04%), error trials (1.14%) and the trial that immediately followed an error (1.14%), as well as outliers, defined as those trials with RTs shorter than 150 ms, larger than 2,000 ms, or straying beyond three standard deviations from the mean, computed on the remaining trials for each participant and block (3.16% of the trials). The results of the ANOVA conducted on RTs are presented in Table 2. The results showed significant effects of *congruency*, indicating that RT was faster for congruent than incongruent trials (644 vs. 801 ms), and of *cueing*, revealing that responses were faster for cued trials (709 vs. 736 ms). There was also a significant *cueing x congruency* interaction, suggesting that the effect of cueing reached significance for congruent trials, *F*_(1, 22)_ = 15.01, *p* < 0.001; $\eta_{\text{p}}^{2}$ = 0.41 (627 vs. 662 ms for cued vs. control trials), whereas it just missed significance for incongruent trials *F*_(1, 22)_ = 3.93, *p* = 0.060; ηp^2^ = 0.15 (791 vs. 811 ms) (see Figure 1A).

**Table 2:** Results of the ANOVAs corresponding to Experiment 1, conducted with reaction times (RT) and percentage of correct responses (Hits%).

**Effect**	***df***	**RT**			**Hits %**		
		***F***	***p***	$\eta_{\text{p}}^{2}$	***F***	***p***	$\eta_{\text{p}}^{2}$
**Exp1: ANOVA congruency** × **cueing** × **order**							
Congruency	1, 22	115.30	< .001^***^	.84	31.68	< .001^***^	.59
× order	1, 22	2.60	.121	.11	0.13	.727	< .01
Cueing	1, 22	9.12	.006^**^	.29	0.62	.441	.03
× order	1, 22	3.09	.093	.12	3.10	.092	.12
Cueing × congruency	1, 22	5.38	.030^*^	.20	0.53	.474	.02
× order	1, 22	1.21	.284	.05	2.05	.166	.09
Order	1, 22	0.20	.657	.01	1.00	.328	.04
**Exp1: ANOVA congruency** × **compliance** × **order**							
Congruency	1, 22	89.46	< .001^***^	.80	14.73	< .001^***^	.40
× order	1, 22	3.80	.064	.15	0.12	.735	.01
Compliance	1, 22	0.33	.569	.02	< 0.01	.929	< .01
× order	1, 22	4.08	.056	.16	0.06	.815	< .01
Compliance × congruency	1, 22	7.03	.015^*^	.24	< .01	.954	< .01
× order	1, 22	1.56	.225	.07	0.07	.797	< .01
Order	1, 22	0.13	.727	< .01	0.01	.929	< .01

The upper panel, using the factor “cueing” refers to the comparison between responses to cueing trials and to those control trials contravening the rules. The lower panel, using the factor “compliance” refers to the comparison between the control trials conforming to or contravening the rules. * < .05, ** < .01, *** < .001.

As for the percentage of hits, we removed the first trial from each block (1.04%) and trials immediately following an error (1.14%). The ANOVA conducted on the remaining trials only produced significant effects of *congruency*, indicating that errors were mainly committed on incongruent trials (the percentages of hits were 99.5 vs. 96.5%, respectively for congruent and incongruent trials). Neither the effect of *cueing* nor the *congruency* × *cueing* interactions were significant in this analysis.

Finally, as for the analyses conducted over the control phase to see whether participants' performance distinguish between trials conforming to or contravening the rules even when the contingencies were not present, the ANOVA conducted on RTs showed no effect of *compliance to the rules* (740 vs. 736 ms) (see Figure 1B). The interaction between *order* and *compliance with the rules* just missed significance, but it showed a pattern suggestive of a numerical advantage in favor of trials conforming to the rules which appeared selectively in participants who completed the control phase after the cueing phase (727 vs. 736 ms), but not in those who were trained in opposite order (752 vs. 737 ms). The analysis also showed a significant interaction between *compliance with the rules* and *congruency*, indicating that congruent responses were faster when they conformed to the rules (651 vs. 662 ms). Intriguingly, this interaction was not modulated by *order*, suggesting that it could be found in performance even before participants were actually exposed to the rules. The analysis on hit percentage only showed significant effects of *congruency*, with more accurate responses for congruent trials (99.5 vs. 96.8%).

### Discussion

Experiment 1 assessed whether reliance on congruency cues conveyed by the preceding trial could be improved by reducing the frequency of high-conflict trials. This hypothesis followed from a strategic view of control regulation, which held that upregulating control would be experienced as more demanding than preparing for a congruent trial, and hence that people would be more willing to act upon those cues if high-demanding trials were relatively scarce. In contrast to this hypothesis, however, the results only showed a non-significant trend to produce faster responses to cued as compared to non-cued incongruent trials, whereas they showed reliable cueing effects for congruent trials. This pattern of results has been repeatedly observed in similar cueing paradigms (Bugg and Smallwood, 2016; Jiménez et al., 2021), and it can be attributed to participants' responding in terms of the distractor whenever they were told that the upcoming trial will be congruent, rather that effectively upregulating control when they were warned about the presentation of an incongruent trial.

These results cast further doubts on the possibility of preparing efficiently for an upcoming conflict on the basis of cues conveyed by the preceding trial. However, one might claim that, rather than reducing the proportion of trials in which the upregulation of control is required, perhaps a better way to improve participants ability to use these cues could be to allow more practice with this setting. Indeed, if one assumes that associative processes play a role in the acquisition of these control regulation responses, then more robust effects could be obtained by providing more practice with these rules, rather than by reducing the opportunity of relying on them, thus contributing to develop an automatic response which could be triggered more efficiently in the presence of the relevant cues (i.e., Braem et al., 2024; Egner, 2014). If this hypothesis is correct, then the manipulation arranged in Experiment 1 may have gone in the opposite direction, as reducing the proportion of incongruent trials would have decreased the practice with incongruency cues.

To explore these two opposite predictions, which arise, respectively, from associative and strategic views of this control cueing process, in Experiment 2 we set up two different versions of the cueing task, comparing the effects observed under conditions of high vs. low proportion of congruency. The condition of high proportion of congruency (HPC) replicated the design arranged in Experiment 1, with conflict arising only in 20% of the trials. In contrast, the condition of low proportion of congruency (LPC) was designed as a complementary setting, in which incongruent trials were presented in 80% of the rials, thus providing more extensive practice with these rules, but also imposing higher cognitive demands to exploit these rules and therefore, according to a strategic view, making it even harder for participants to exploit these rules.

## Experiment 2

Experiment 2 was designed as a replication and extension of Experiment 1, manipulating the proportion of congruency in two independent groups, one replicating the HPC conditions arranged in Experiment 1, and the other receiving the opposite conditions of LPC, which inverted the rates of congruent and incongruent trials. Our aim was to test whether reliance on high-conflict cues could be impaired, facilitated, or not affected by increasing the opportunities of practicing the rules.

### Method

#### Participants, apparatus and stimuli

Forty-eight different participants from the same population of Experiment 1 (41 female, M_age_ = 20.35, range = 18–24) were randomly assigned to HPC or LPC conditions, creating two groups of 24 participants each. The apparatus and stimuli were the same as described for Experiment 1. Participants in the HPC were presented with an exact replication of the previous experiment, in which congruent trials arose in 80% of the trials, and incongruent trials were presented in the remaining 20% of the trials. The LPC condition inverted these proportions, so that participants responded to congruent trials in 20% of the trials, and to incongruent trials in the remaining 80% of the trials. For each participant, the colors designated as cues for the more frequent type of successor were chosen more often than the remaining two colors, and these proportions were maintained over the control phase, even though the predictive contingencies were removed.

#### Procedure and design

The procedure and design were analogous to that described for Experiment 1, with the exception that Experiment 2 involved an additional between-participants factor corresponding to *proportion of congruency* (HPC vs. LPC).

### Results

As in Experiment 1, the analysis of RTs was conducted after excluding the first trial of each block (1.04%), errors (1.14 %), the trial that immediately followed an error (1.12%), and outliers (2.13% of the trials). The results of the ANOVAs are summarized in Table 3. The ANOVA conducted on the remaining RTs showed no main effects of *order*, or *proportion of congruency*. The expected effect of *congruency* was significant (648 vs. 765 ms, respectively, for congruent and incongruent trials), and the results also showed the typical list-wide proportion of congruency effect, as inferred from the *congruency* × *proportion of congruency* interaction. This showed that congruency effects were larger for the HPC group (628 vs. 804 ms) than for the LPC group (668 vs. 726 ms). The effect of *cueing* was not significant (700 vs. 713 ms, respectively for cued and control trials), but there was a significant *congruency x cueing* interaction, as well as a significant three-way *congruency* × *cueing* × *proportion of congruency* interaction which merit specific analysis.

**Table 3:** Results of the ANOVAs corresponding to Experiment 2, conducted with reaction times (RT) and percentage of correct responses (Hits%).

**Effect**	***df***	**RT**			**Error %**		
		***F***	***P***	$\eta_{\text{p}}^{2}$	***F***	***p***	$\eta_{\text{p}}^{2}$
**Exp2: ANOVA congruency** × **cueing** × **order** × **PC**							
Congruency	1, 44	220.06	< .001^***^	.83	24.39	< .001^***^	.36
× PC	1, 44	55.42	< .001^***^	.56	9.83	.003^**^	.18
× order	1, 44	0.01	.911	< .01	0.24	.627	.01
× PC × order	1, 44	2.65	.111	.06	2.13	.151	.05
Cueing	1, 44	3.02	.089	.06	0.44	.513	.01
× PC	1, 44	0.03	.875	< .01	0.15	.702	< .01
× order	1, 44	9.80	.003^**^	.18	1.14	.291	.03
× PC × order	1, 44	0.20	.661	< .01	0.73	.398	.02
Cueing × congruency	1, 44	15.57	< .001^***^	.26	2.70	.108	.06
× PC	1, 44	39.45	< .001^***^	.47	0.32	.575	.01
× order	1, 44	0.48	.492	.01	0.07	.794	< .01
× PC × order	1, 44	0.83	.368	.02	0.74	.394	.02
PC	1, 44	0.45	.504	.01	3.07	.087	.07
× order	1, 44	0.75	.390	.02	1.29	.263	.03
Order	1, 44	1.26	.267	.03	0.11	.742	< .01
**Exp2: ANOVA congruency** × **compliance** × **order** × **PC**							
Congruency	1, 44	233.18	< .001^***^	.84	19.52	< .001^***^	.31
× PC	1, 44	63.23	< .001^***^	.59	8.02	.007^**^	.15
× order	1, 44	0.56	.460	.01	0.81	.373	.02
× PC × order	1, 44	2.92	.094	.06	3.23	.079	.07
Compliance	1, 44	5.51	.023^*^	.11	0.40	.529	.01
× PC	1, 44	4.65	.037^*^	.10	0.18	.670	< .01
× order	1, 44	3.13	.084	.07	0.76	.389	.02
× PC × order	1, 44	1.61	.211	.04	< .01	.953	< .01
Compliance × congruency	1, 44	7.40	.009^**^	.14	1.05	.312	.02
× PC	1, 44	53.67	< .001^***^	.55	0.31	.583	< .01
× order	1, 44	0.98	.328	.02	0.78	.383	.02
× PC × order	1, 44	0.49	.488	.01	0.02	.890	< .01
PC	1, 44	1.17	.285	.03	4.56	.038^*^	.09
× order	1, 44	0.30	.586	< .01	2.08	.157	.05
Order	1, 44	< .01	.994	< .01	0.16	.694	< .01

PC stands to proportion of congruency. The upper panel, using the factor “cueing” refers to the comparison between responses to cueing trials and to those control trials contravening the rules. The lower panel, using the factor “compliance” refers to the comparison between the control trials conforming to or contravening the rules. * < .05, ** < .01, *** < .001.

**Figure 2:**
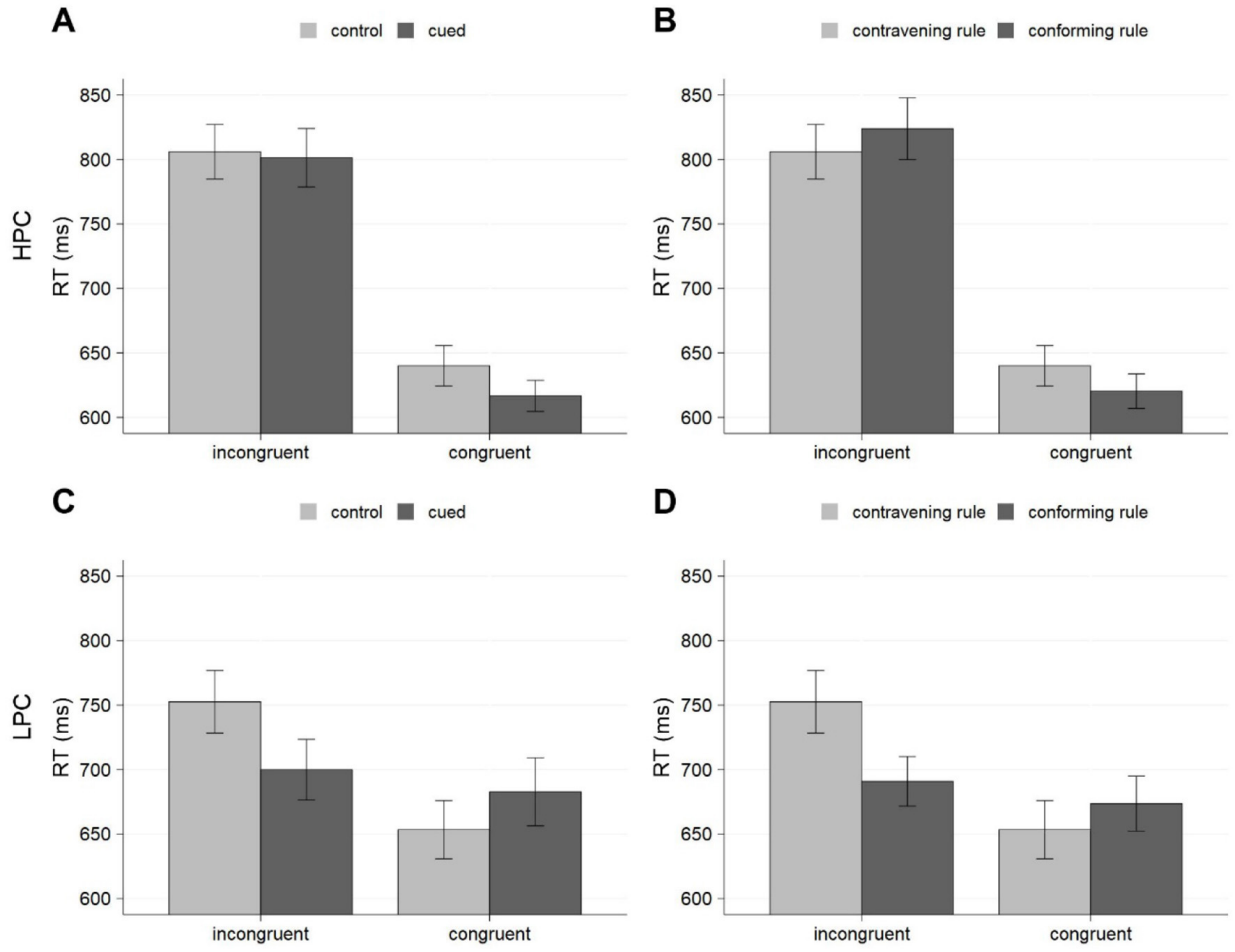
Mean RTs for cueing effects **(A, C)** and the presence vs. absence of the predictive rule on control blocks **(B, D)** for Experiment 2, separately for HPC **(A, B)** and LPC **(C, D)**. Error bars represent the standard error of the mean.

Separated analyses conducted for HPC and LPC conditions to explore the three-way interaction indicated that, in the HPC group, which reproduced the conditions of Experiment 1, neither the effect of *cueing, F*_(1, 22)_ = 1.53, *p* = 0.230; $\eta_{\text{p}}^{2}$ = 0.07, nor the *cueing* x *congruency* interaction reached significance, *F*_(1, 22)_ = 2.08, *p* = 0.163; $\eta_{\text{p}}^{2}$ = 0.09, even though the numerical pattern resembled that observed in the previous experiment (see Figure 2A). Thus, the results seemed noisier, but the pattern was similar to that found in Experiment 1, showing a larger numerical advantage in favor of cued trials for the congruent trials (617 vs. 640 ms) and a smaller difference between them in incongruent trials (801 vs. 806 ms). As for the LPC condition, the main effect of *cueing* was again non-significant, *F*_(1, 22)_ = 1.51, *p* = 0.232; $\eta_{\text{p}}^{2}$ = 0.06, but there was a significant *cueing* × *congruency* interaction, *F*_(1, 22)_ = 75.56, *p* < 0.001; $\eta_{\text{p}}^{2}$ = 0.77, which revealed a very contrastive pattern (see Figure 2C). In this case, the cueing effect was significant for incongruent trials [700 vs. 753 ms, *F*_(1, 23)_ = 19.43, *p* < 0.001; $\eta_{\text{p}}^{2}$ = 0.46]. For congruent trials, it produced an opposite trend to respond slower for cued than for control trials [683 vs. 653 ms, *F*_(1, 23)_ = 6.40, *p* = 0.019; $\eta_{\text{p}}^{2}$ = 0.22].

As for the hit rates, the corresponding ANOVA showed a significant effect of *congruency*, indicating that more correct responses were given to congruent than incongruent trials (99.4 vs. 97.1%), and a significant *congruency* × *proportion of congruency* interaction, indicating that the effect of congruency was present on the HPC group (99.6 vs. 95.8%) but not on the LPC group (99.2 vs. 98.4%). However, neither the effect of *cueing* nor any interaction involving *cueing* reached significance.

Finally, as for the analysis conducted exclusively on the control trials to assess whether participants responded differently to these trials in terms of whether they complied or failed to comply with the predictive rules, this confirmed the unexpected results found in Experiment 1. Focusing on the effects and interactions involving *compliance with the rules*, we found a significant effect of this factor, indicating that participants responded faster to trials consistent with the rules even on these control blocks, in which the contingencies were absent (702 vs. 713 ms). Moreover, this factor interacted with *congruency*, showing that the advantage of conforming to the rules was observed for incongruent (757 vs.779 ms) but not for congruent (647 vs. 647 ms) trials. There was also a *compliance* × *proportion of congruency* interaction, suggesting that the advantage of conforming to the rules was specifically obtained in the LPC group (682 vs. 703 ms) but not in the HPC condition (722 vs 723 ms). Finally, there was a significant three-way interaction involving *compliance, congruency*, and *proportion of congruency* which deserved further investigation.

Specific analyses conducted for each group showed that, in the HPC group, the pattern replicated that obtained in Experiment 1, showing a cross-over interaction between *congruency* and *compliance with the rules* (see Figure 2B), *F*_(1, 22)_ = 8.71, *p* = 0.007; $\eta_{\text{p}}^{2}$ = 0.28. Thus, participants responded faster to congruent trials complying with the rules than to those opposite to these rules (620 vs. 640 ms), but for incongruent trials they showed the inverse tendency, producing faster responses to those trials which failed to obey the rules rather than to those which agreed with the rules (824 vs. 806 ms). In contrast, the effect of *compliance with the rules* was significant for the LPC group, *F*_(1, 44)_ = 8.35, *p* = 0.009; $\eta_{\text{p}}^{2}$ = 0.28, as well as its interaction with *congruency, F*_(1, 22)_ = 64.47, *p* < 0.001; $\eta_{\text{p}}^{2}$ = 0.75, showing a significant advantage of conforming to the rules on incongruent trials (691 vs. 753 ms), but a numerical disadvantage on congruent trials (674 vs. 653 ms.; see Figure 2D). Finally, three-way interaction involving *order, congruency*, and *compliance with the rules* was not significant, *F*_(1, 22)_ = 0.05, *p* = 0.819; $\eta_{\text{p}}^{2}$ < 0.01, thus suggesting that the above-mentioned pattern observed over the control blocks did not depend on the previous experience with the rules, as it arose regardless of whether those control blocks were scheduled before or after the cueing phase.

### Discussion

The results of Experiment 2 showed the standard effects of congruency and list-level proportion of congruency, but they showed no clear cueing effects, neither in the conditions that mirrored those arranged in Experiment 1 (i.e., the HPC group), nor in those in which participants were trained more extensively with incongruent trials. The HPC condition showed a pattern analogous to that found in Experiment 1, in that cueing effects were numerically larger for congruent than for incongruent trials, although in this case the results were a bit noisier. In contrast, the LPC condition showed cueing effects exclusively for incongruent trials, as it could be predicted if greater levels of practice with cued trials could improve participants' ability to exploit high-conflict cues. However, the unexpected pattern observed in the analysis of the control phase, which suggests that participants in this group responded faster to cued incongruent trials even over the control phase, when the cues were not informative, and even if they completed the control phase before the cueing phase, revealed that these findings should be due to an effect independent from training with the cueing conditions.

A potential account for the differences observed between responding to control trials conforming and not conforming to the rules might be attributed to the specific frequencies with which each type of trial is presented over the whole procedure. Indeed, the procedure strived to maintain a homogeneous structure over both cueing and control phases, maintaining the same proportion of congruency and the same unbalanced distribution of the four colors, determined by their values as congruency cues. Thus, for instance, participants in the HPC group experienced congruent trials in 80% of the trials, and they responded to those colors designated as cues for such congruent successors more often than to those assigned as predictors of incongruent trials. Because of this unbalanced distribution of colors, one might expect that participants would not only respond faster to the most frequent colors, but also that they respond slower to those trials coming after a relatively odd color. The slowdown of responding after infrequent trials is consistent with the oddball effect (Barcelo et al., 2006), and it has been alleged as a potential cause of the phenomenon of post-error slowing (Notebaert et al., 2009), which attributes the delay observed after an error to the infrequent character of errors. In the present conditions, if a slowdown of responding was produced after reacting to a relatively odd trial, this effect could be confounded with a cueing effect obtained selectively in response to the most frequent trials. Specifically, in the HPC condition, if both congruent trials and their color predictors arise more likely than their counterparts, this would produce faster RTs when the congruent trials arose after their correct predictors, which are also the most frequent colors, and slower when the same congruent trials arose after the less frequent colors designated as predictors of incongruent successors. In contrast, for the LPC group, if both incongruent trials and their predictors are more likely, this would produce faster RTs when incongruent trials arose after the common colors assigned as predictors of such high-conflict trials, and slower RTs when they arose after the relatively odd colors assigned as predictors of congruent successors. Importantly, if that effect is provoked by the relative frequency of the predictors, rather than by their predictive value, they should be observed over both the cueing and control phases, regardless of the order of presentation of these phases, and regardless of whether the predictive contingencies are operative.

The pattern of results described in the above paragraph corresponded exactly with that found over the control phase in Experiments 1 and 2, and therefore indicate that the main comparison from which we inferred learning could be affected by the same confound, thus revealing a tendency to respond faster after a frequent trial, rather than the results of the cueing effects. Under these circumstances, we must conclude that the previous designs may contain a confounding factor that precludes a clear interpretation of their results in terms of the effects of cueing. Even though these experiments produced a serendipitous finding about the generalization of the oddball effect (Barcelo et al., 2006) to a Stroop task in which certain colors were less frequent, they have not led us closer to the goal of building up the boundary conditions of congruency cueing. The results indicate that, in order to explore whether reducing the proportion of high-conflict trials could increase participants' reliance on congruency cues, it is necessary to maintain the balance between all possible colors and responses, so that the effect of cueing would not become confounded with more elementary tendencies such as that of increasing the delay after responding to an odd trial.

With these ideas in mind, the two final experiments of the series were designed to explore the original hypothesis that reducing the exposition to the most demanding high-conflict cues could improve their efficacy, either by making these occasions more salient, or by reducing the accumulated effort invested in following these rules. Instead of using two less frequent colors as cues for incongruent trials, as we did in the previous experiments, in Experiment 3 and 4 we generated all four colors with the same likelihood but designated a single color as predictive of incongruent successors. In Experiment 3, the remaining three colors were predictive of congruent successors, thus producing conditions of HPC like those used in Experiment 1. In contrast, in Experiment 4 we arranged a more balanced design, using another single color as predictor of congruent successors, and the two remaining colors as predictors of neutral trials, in which the color words were replaced by a string of “xxxxx”. With this design, we were able to compare the use of cues predictive of congruent and incongruent successors under comparable conditions. In addition, as we included trials in which the color cue was conveyed by non-conflict trials, we could also assess whether the effect of congruency cues could be observed more easily when the cueing trial does not require control, and hence does not compete with the resources demanded by preparing for an upcoming conflict. Because these two experiments were otherwise analogous, we described their designs and results conjunctly.

## Experiments 3 and 4

### Method

#### Participants, apparatus and stimuli

Twenty-four different participants from the same population of the previous experiments were assigned to each of these experiments. Fifteen female and nine male participants, M_age_ = 32.75, range = 20–56, were assigned to Experiment 3, and 12 female and 12 male participants, M_age_ = 24.92, range = 19–35, completed Experiment 4. The apparatus and stimuli were analogous to those described for the previous experiments, with the following exceptions. First, each color was presented randomly, and therefore they all arose with the same likelihood. Second, during the cueing phase only one color was designated as predictor of high-conflict successors for each participant, thus forcing the proportion of incongruent trials to be .25. The assignment of colors to their predictive value was counterbalanced, so that each color acted as a conflict cue for the same number of participants. In Experiment 3, the remaining three colors were all followed by congruent successors, thus producing a proportion of congruent trials of .75. In Experiment 4, congruent trials were cued by a single color, equally counterbalanced between participants, and hence they also arose in 25% of the trials. The remaining 50% of the trials were preceded by the other two colors, and they were arranged as neutral trials composed by a colored string of “xxxxx”. During the control phase all predictive contingencies were removed, but the proportions of each type of trials were maintained. The order of presentation of cueing and control phases were also counterbalanced between participants.

#### Procedure and design

The procedure was analogous to that described for the previous experiments, with the exception that we changed the *Inquisit* procedure selected to encode vocal responses. Instead of relying on the speech recognition modules to recognize responses and to give online feedback to participants, which might produce delays in the progression of some trials when responses were not successfully recognized, we moved to a voice-record tool, which uses the voice key to measure participants' RT, and records each response without further analyses, thus reserving the analysis to an offline procedure, which was primarily based on the automatic tool provided by the program, and was then exhaustively supervised by human encoders. This precluded the possibility of giving feedback online during participants' performance, but in exchange it ensured a more even presentation. Trials with no response were timed out 2,500 ms after the presentation of the target.

As for the experimental design, this was analogous to that used for Experiment 1. However, because in Experiment 4 we also arranged neutral trials in 50% of the trials, we included an additional analysis to address the question of whether congruency cueing could work better on those trials cued by neutral trials, rather than on those cued by either congruent or incongruent trials.

### Results

**Figure 3:**
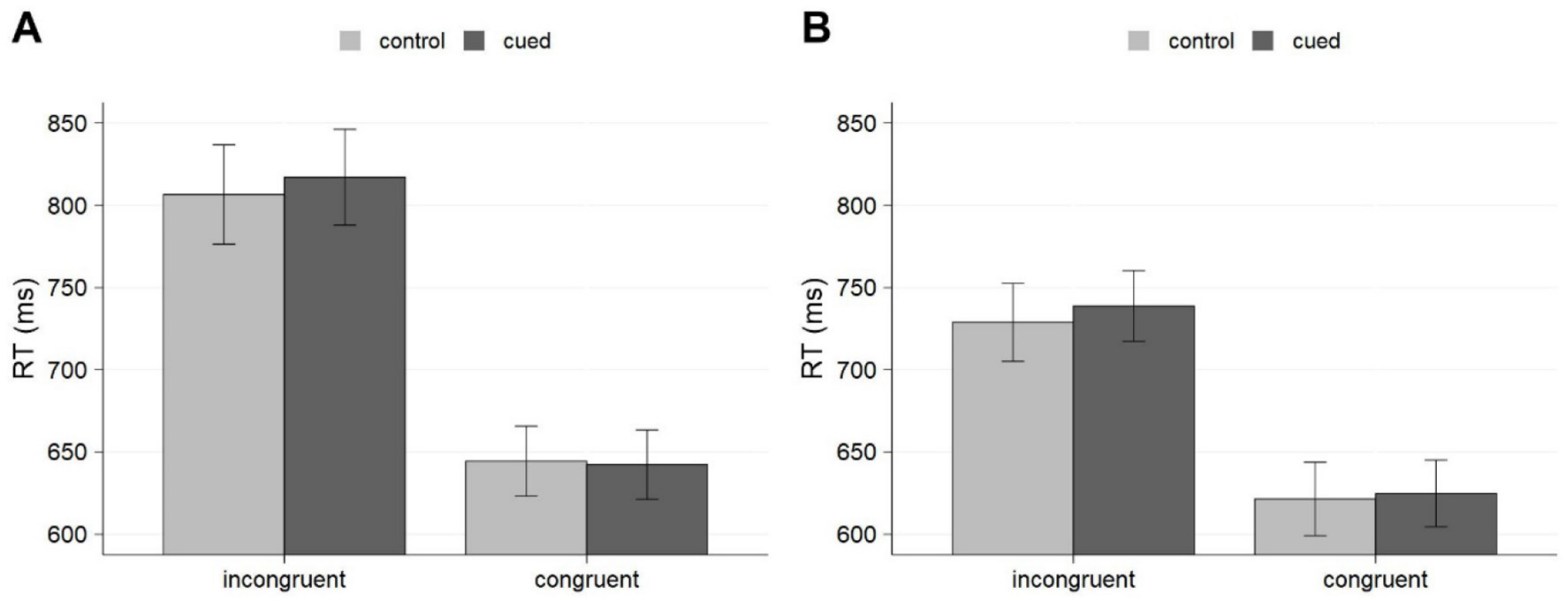
Mean RTs for the cueing effects on Experiment 3 **(A)** and 4 **(B)**. Error bars represent the standard error of the mean.

For Experiment 3, the analysis of RTs was conducted after excluding the first trial of each block (1.04%), errors (2.5%), the trial that immediately followed an error (2.38%), and outliers (1.79% of the trials). For Experiment 4, the same trimming procedure excluded also a 1.04% of the trials as the first trial on each block, another 2.5% of the trials as errors, 2.38% of trials as immediately following an error, and 2.17% of trials as outliers. The results of the overall ANOVAs conducted on RTs and percentage of correct responses are presented on Table 4. The analysis conducted on RTs showed no main effects of *order*, neither in Experiment 3, nor in Experiment 4. The expected effect of *congruency* was significant in both experiments (643 vs. 812 ms for congruent and incongruent trials in Experiment 3 and 623 vs. 734 ms in Experiment 4). There was no evidence for an effect of *cueing* or a significant *congruency x cueing* interaction in none of these experiments (see Figure 3). Finally, Experiment 4 also allowed to assess the impact of cueing selectively in those trials in which the cue was conveyed through a non-conflict, neutral trial. The results were analogous to those described for the whole set: there was only a significant effect of *congruency* (626 vs. 736 ms), but not a main effect of *cueing* (682 vs. 680 ms), nor a significant *cueing* × *congruency* interaction.

**Table 4:** Results of the ANOVAs from Experiments 3 and 4, conducted with reaction times (RT) and percentage of correct responses (Hits%).

**Effect**	***df***	**RT**			**Error %**		
		***F***	***P***	$\eta_{\text{p}}^{2}$	***F***	***p***	$\eta_{\text{p}}^{2}$
**Exp3: ANOVA congruency** × **cueing** × **order**							
Congruency	1, 22	108.88	< .001^***^	.83	97.04	< .001^***^	.82
× order	1, 22	0.91	.351	.04	0.30	.588	.01
Cueing	1, 22	0.32	.578	.01	1.28	.270	.06
× order	1, 22	2.28	.145	.09	0.02	.904	< .01
Cueing × congruency	1, 22	3.72	.067	.15	1.17	.290	.05
× order	1, 22	4.92	.037^*^	.18	0.02	.887	< .01
Order	1, 22	0.67	.422	.03	0.25	.620	.01
**Exp4: ANOVA congruency** × **cueing** × **order**							
**Full dataset**							
Congruency	1, 22	129.17	< .001^***^	.85	35.40	< .001^***^	.62
× order	1, 22	1.78	.196	.08	0.03	.860	< .01
Cueing	1, 22	0.25	.623	.01	0.78	.386	.03
× order	1, 22	0.30	.591	.01	0.10	.758	< .01
Cueing × congruency	1, 22	0.64	.432	.03	0.46	.506	.02
× order	1, 22	0.08	.780	< .01	0.06	.817	< .01
Order	1, 22	0.07	.794	< .01	0.49	.817	< .01
**Neutral trials as cues**							
Congruency	1, 22	88.14	< .001^***^	.80	24.43	< .001^***^	.53
× order	1, 22	0.98	.334	.04	< .01	.947	< .01
Cueing	1, 22	0.02	.889	< .01	0.04	.846	< .01
× order	1, 22	0.53	.474	.02	0.10	.757	< .01
Cueing × congruency	1, 22	0.63	.436	.03	0.03	.896	< .01
× order	1, 22	0.57	.458	.03	0.30	.592	.01
0 Order	1, 22	0.11	.745	< .01	0.04	.838	< .01

In Experiment 4, the ANOVAs were conducted twice, first considering all trials as cues, and then selecting only those trials preceded by a neutral trial. * < .05, ** < .01, *** < .001.

As for the hit rates, we removed from the analyses the first trial of each block (1.04% for both experiments) and trials after an incorrect response (2.53% on Experiment 3, 2.48% on Experiment 4). The ANOVAs on the whole dataset showed only significant effects of *congruency* in both experiments (99.5 vs. 92.2%, respectively for congruent and incongruent trials for Experiment 3, and 99.4 vs. 92.7% for Experiment 4). No other effect or interaction approached significance in any of these analyses. The analysis conducted for Experiment 4 specifically on trials presented after neutral trials reproduced the same pattern, showing only an effect of *congruency*, and no hint of an effect or interaction involving cueing (*F*s < 1).

### Discussion

Experiments 3 and 4 were designed to test a hypothesis that followed from a strategic view of regulating control: Assuming that preparing for a high-conflict trial is more demanding than preparing for a congruent trial, and that the use of congruency cues depends on a strategic decision based on its expected value (Shenhav et al., 2013), we predicted that congruency cues should work better in conditions in which high-conflict trials were relatively scarce. Consequently, these two experiments arranged conditions in which high-conflict trials were presented in only a quarter of the trials, using either congruent trials for the remaining 75% of the trials (in Experiment 3) or combining a 25% of congruent trials and a 50% of neutral trials (Experiment 4). In both cases, we compared performance in cued conditions, in which participants were informed about the predictive value of the preceding colors, against a control phase in which such cues were not valid. However, we found no evidence indicating that participants could take advantage of these explicit cues to respond more efficiently to cued trials. Importantly, in Experiment 4 we were able to assess whether such cueing effects could be observed better when the cue was conveyed by neutral trials, to preclude the potential conflation provoked by the coincidence of two simultaneous requirements of control, one concerning the demands imposed by current trial, and another involving the proactive information conveyed by each trial regarding its successor. However, even in these conditions we found no evidence that participants could use the cues provided in the preceding trial to get better prepared to respond to the amount of conflict expected on the following trial.^1^

## General discussion

The aim of this research was to investigate the dynamics of cognitive control, assessing whether congruency cues explicitly conveyed by the preceding target can be exploited by participants in an interference task to deal more efficiently with a cued conflict. Starting from the cueing effects obtained when the cues were included in the interval between successive trials (Bugg and Smallwood, 2016; Jiménez et al., 2020, 2021), we aimed to generalize this effect to conditions in which the cueing information was conveyed by the preceding trial. Even though participants were informed about the cueing value of the preceding targets and were necessarily attending and responding to those informative targets, previous studies failed to obtain evidence of this cueing effect when high- and low-conflict trials were balanced. In the present study we reasoned that, if control regulation depends on a strategic decision based on its expected value (Shenhav et al., 2013), and if upregulating control incurs larger costs than downregulating it, then decreasing the proportion of high-conflict trials could minimize these costs, and correspondingly increase the likelihood that participants adopt the strategy of relying on the cues.

In Experiment 1, we failed to see a clear advantage for responding to cued as compared to non-cued incongruent trials, even though there was a trend in this direction, and the results suggested that participants might be using the cues to respond faster to cued congruent trials. In Experiment 2 we compared cueing effects in conditions of high vs. low proportion of congruent trials (HPC vs. LPC), and the results were supportive of the opposite hypothesis, as they showed that cueing effects were selectively observed for incongruent trials in the LPC group, when participants were more frequently exposed to high-conflict trials. However, a detailed analysis of the responses emitted over the control blocks revealed that these effects were most probably due to an artifact coming from the biased frequency of each color, as they were also observed over the control phase, when the sequential contingencies were absent, and regardless of the order in which control and cueing phases were scheduled. Arguably, this pattern of results should be attributed to a confound caused by the low frequency with which the colors designated as predictors of the less frequent type of conflict were presented over the whole procedure, which produced a delay in response to those trials that followed these relatively odd trials (Barcelo et al., 2006). In Experiment 1, and in the HPC condition from Experiment 2, because the less frequent colors were those designated as predictors of high-conflict trials, the slowdown of responding to congruent trials coming after a color predictive of an incongruent successor could be wrongly interpreted as revealing a cueing effect arising selectively for congruent trials. In contrast, in the LPC condition from Experiment 2, when the infrequent trials were those assigned as predictors of a low-conflict trial, this would tend to produce slower RTs in response to incongruent trials when they come after a color predictive of a congruent successor, and thus could be interpreted as an effect of cueing produced selectively for incongruent trials. Importantly, the fact that both effects were also observed over the control blocks, regardless of the order of presentation of these blocks, preclude the interpretation of these results as showing a genuine cueing effect.

Experiments 3 and 4 were arranged to avoid the confound revealed by the former analyses, thus presenting all four colors with the same likelihood, but maintaining low levels of conflict, and using only one color as predictive of incongruent successors. The remaining three colors were either used as cues for congruent successors, in Experiment 3, or as predictors of congruent and neutral trials, reserving one of them as predictive of congruent successors, and the remaining two colors as predictive of neutral trials (Experiment 4). This allowed us to explore whether cueing effects were easier to obtain after a neutral trial, as it could be expected if part of the difficulties to exploit the cueing information depends on the conflation between the control demands required by the cueing trial and the information conveyed by the cue regarding the conflict expected on the successor. The results clearly showed that such cueing effects were absent in both experiments, thus confirming the difficulty of exploiting these congruency cues on a trial-by-trial basis.

The results of this series of experiments allow three main conclusions. First, we found no evidence of congruency cueing in a Stroop task despite using explicit instructions, arranging completely reliable contingencies between the previous target and the congruency of the successor, and reducing the number of high-conflict trials to decrease the demands made by continuously exploiting incongruency cues. This holds true at least for the practice allowed within a single session of training which involves over 50 pairings between each cue and its predicted outcome. These negative results contrast with a few positive results obtained in previous paradigms when the cue was conveyed by an additional stimulus located in the interval between successive trials (Bugg and Smallwood, 2016; Jiménez et al., 2021, Experiment 8). In the latter of these studies, the shape of the fixation point was arranged as the congruency cue, and its duration was the same as the fixation point used in the present study. Thus, if participants could use the identity of their previous response as a cue, they would have the same time as in the previous study to encode the value of the cue and to prepare for the upcoming conflict. If, on the other hand, they could exploit the color of the preceding trial as a cue, they would have a slightly larger interval, including their RT, in addition to the RSI, to prepare for the predicted conflict. The fact that we found no advantage of the cue in these conditions indicates that trial-by-trial cueing is more difficult than presenting the cue in the interval between successive trials.

The results of the present study are also in contrast with the speed and efficiency with which other contingency relations seem to affect control regulation when the cues are presented simultaneously with the target dimension, as it occurs in the item-specific (Schmidt and Besner, 2008), or in the context-specific congruency effects (Bugg et al., 2022; Schmidt and Lemercier, 2019). To reconcile both literatures, one should probably assume either that timing is the key, and that reactive control is more easily affected by learning than proactive effects, or that the effect provoked by synchronic cues could be partially attributed to other effects different from learning, such as those caused by the retrieval of the last episode reproducing the same cue-target ensemble (Giesen et al., 2020). There is evidence showing that the effect of episodic matching is significant in these paradigms (Gallego et al., 2023; Rothermund et al., 2022), but there is also evidence suggesting that some learning effects remain even after the impact of episodic effects is controlled, for instance when the context effects are measured on diagnostic trials, which are free from episodic factors. Thus, if learning effects have an independent impact on the regulation of control in synchronic preparations, this reinforces the conclusion that a boundary condition for the impact of learning on control regulation has to do with the sequential nature of the cues arranged in this cueing paradigm.

Second, the results provided by Experiment 4 also allowed us to conclude that the origin of the difficulty to obtain benefits from trial-by-trial cueing does not come from the merge of the control demands made by the cueing trial and the control information conveyed by that trial on the conflict expected for the following trial. If this conflation was responsible for the lack of effects obtained in this paradigm, one should expect to obtain neater cueing effects on trials cued by neutral trials, in which the internal conflict would be absent. In contrast to this prediction, the results of this experiment provided no indication of this effect.

Finally, from a methodological point of view, our results highlight a potential confound introduced by our sequential manipulation of the cues, in conditions like those arranged in Experiments 1 and 2, where the likelihood of the cued dimension differs from that expected by chance. In these cases, because the cued dimension determines the frequency of the cues, this produces an unbalanced distribution of the cues, which may produce some unexpected effects which can become confounded with the measures of interest. Fortunately, the arrangement of a control condition that mirrored the same frequencies arranged over the cueing phase allowed us to detect that the effects obtained in the comparison between the cueing phase and those trials from the control phase that failed to comply with the rules could also be obtained within the control phase, by comparing responses to trials complying or failing to comply with the cueing rules. This latter effect pointed to a factor different from the contingencies manipulated over the cueing phase, which was identified as a delay provoked selectively in those trials that come immediately after an odd trial. This phenomenon resembles the oddball effect (Barcelo et al., 2006), and it points to a more general effect that would arise not only after external and irrelevant events, or after committing an error in response to a task in which errors are infrequent, as proposed by Notebaert et al. (2009), but also after any task-relevant but relatively infrequent trial, as it occurs in this case with the colors that were presented less frequently in a Stroop task.

## Limitations and conclusions

The results of this study contribute to highlight the difficulties of regulating control proactively on a trial-by-trial basis, even when this regulation could be based on explicit and reliable cues conveyed by the previous response, without requiring the processing of additional signs, and in contexts in which only a reduced proportion of trials requires an upregulation of control. The extent to which the observed difficulties can be generalized to other interference paradigms, different temporal conditions or different motivational manipulations is currently unknown and deserves further investigation. However, the difficulty repeatedly observed in these trial-by-trial manipulations stands in contrast with some positive results reported in analogous paradigms where the cues are located in the interval between successive trials (Jiménez et al., 2021). Further research is needed to better understand why the boundary between successive trials does hinder the exploitation of these sequential cues, and under which conditions could they be successfully crossed.

## Data Availability

The datasets presented in this study can be found in online repositories. The names of the repository/repositories and accession number(s) can be found at: Open Science Foundation (https://osf.io/xgkjr).

## Footnotes

^1^Additional exploratory analyses were conducted on RTs in these two experiments, which were free of frequency confounds, to ascertain whether the cueing effects could be better exploited after a congruent trial rather than after an incongruent trial, assuming that less conflict trials would provide better opportunities to exploit the cues. However, these analyses provided no evidence supporting that conclusion, as the *Cueing* × *Previous Congruency* interaction did not reach significance in any of them. In Experiment 3, the observed trend *F*_(1, 22)_ = 3.81, *p* = 0.06; $\eta_{\text{p}}^{2}$ = 0.15 pointed in the opposite direction, indicating that no cueing effect was found after congruent trials (726 vs. 725 ms), and numerically slower RT were obtained in response to cued as compared to control trials after incongruent trials (743 vs. 728 ms). The analysis conducted on Experiment 4 showed almost null results, *F*_(1, 22)_ < 0.01, *p* = 0.99; $\eta_{\text{p}}^{2}$ < 0.01.
